# Supplementary material for: Deep Learning Analysis for Predicting Tumor Spread through Air Space in Early-Stage Lung Adenocarcinoma Pathology Images
Source: Cancers (Basel). 2024 Jun 3;16(11):2132. doi: 10.3390/cancers16112132 (PMC11172106; doi:10.3390/cancers16112132)
Supplement: Supplementary file 1 [file cancers-16-02132-s001.zip › cancers-2996689-supplementary.pdf]

Supplementary Information

Table S1. A comprehensive review of recent studies on predicting spread through air spaces (STAS) using CT imaging.

Paper Review							
	Study	Year	Condition of Lesion	Multi-center	Training/Testing	Method	Performance (internal/external validation)
	Jiang et al. [1]	2020	--	No	195/84	Radiomic features+Age+random forest	AUC=0.75(external)
	Chen et al. [2]	2020	Stage I	No	233/112	Radiomic features+Naïve Bayes	AUC=0.69 (external)
	Zhuo et al. [3]	2020	--	No	149/63	Radiomics features+nomogram	AUC=0.99 (external)
	Li et al. [4]	2020	--	Yes	462/116	Radiomics features+logistic regression	AUC=0.69(external)
**Radiomic-based methods**	**Our past study [5]**	2021	--	Yes	277/100	Radiomics features+patch-wise prediction	AUC=0.83 (external)
	Onozato et al. [6]	2021	Size < 2 cm	No	226/--	Radiomics features+XG-boost	AUC=0.77 (internal)
	Liao et al. [7]	2021	Stage I	No	171/85	Radiomics features+nomogram	AUC=0.87 (external)
	Bassi et al. [8]	2022	--	No	99/50	Radiomics features+Naıve Bayes+k-Nearest Neighbors+Random Forest+Logistic Regression	AUC=0.66 (external)
	Takehana et al. [9]	2022	--	No	203/136	Radiomics features+logistic regression	AUC=0.76 (external)

	Gong et al. [10]	2023	--	No	430/107	Radiomics features+linear regression	AUC=0.80 (external)
	Suh et al. [11]	2024	Stage I	No	301/158	Radiomics features	AUC=0.87 (external)
**Deep learning -based methods**	Tao et al. [12]	2022	--	No	153/50	3D CNN	AUC=0.80 (external)
	**Our past study [13]**	2024	C/T ratio<0.5 ; size<3 cm	Yes	458/123	3D CNN	AUC=0.82 (external)
	Wang et al. [14]	2024	Solid or Part-Solid	Yes	512/90	3D CNN	AUC=0.81(external)

3D CNN: three-dimensional convolutional neural network model.

Table S2. review and synthesis of literature on histopathological images.

Paper Review							
**Deep learning-based methods**	Study	Year	Condition of Lesion	Problem Type	Patients numbers	Method	Performance (internal/external validation)
	Elazab et al. [15]	2024	Brain tumor	Detecting+ Classification	870	YOLOv5+ResNet50	AUC=0.98(external)
	Tsuneki et al. [16]	2022	multi-organ adenocarcinoma	classification	8896	EfficientNetB1	AUC=0.98(external)
	Kanavati et al. [17]	2022	Breast ductal carcinoma in situ	classification	1382/548	CNN+RNN	AUC=0.96(external)/ AUC=0.98(external)
	Lu et al. [18]	2022	breast cancer	predict	589	GNN	AUC=0.80(external)
	Kim et al. [19]	2024	lung adenocarcinoma	predict	164	multiple instance learning	AUC=0.68(external)
	Shim et al. [20]	2023	STAS	Identifying	393	CNN	AUC=0.77(external)/ AUC=0.76(external)

	Wang et al. [21]	2022	bone marrow cell	Detection+ classification	12,426	R-CNN	AUC=0.99(external)
	Abdeltawab et al. [22]	2022	kidney	classification	64	ResNet18	AUC=0.92(external)
	Ding et al. [23]	2023	Breast Cancer	Segmentation	20000	supervised training	AUC=0.82(external)
	Chen et al. [24]	2022	cervical cancer	predict	251	Transfer learning	AUC=0.80(external)
	Zhang et al. [25]	2022	pancreatic masses	segmentation	194	DCNN	AUC=0.97(external)
	Zhao et al. [26]	2023	Digital Pathology Images	Segmentation	476	RGSB-UNet.	AUC=0.83(external)
	Su et al. [27]	2023	esophageal mucosa and squamous cell neoplasm	Segmentation	10	U-Net	AUC=0.96(external)
	Zhang et al. [28]	2024	early gastric cancer diagnosis	Segmentation	378	VENet	AUC=0.90(external)

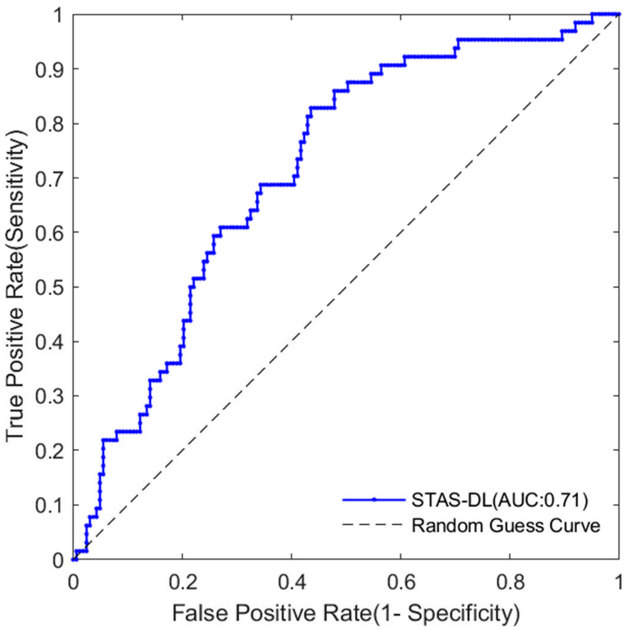

Figure S1. Receiver operating characteristic curves (ROC) for STAS prediction by the without the False Positive Reduction step in the testing cohort (n = 227)

Table S3. Performance of without the False Positive Reduction step of STAS detection model.

Methods	Accuracy (%)	Sensitivity (%)	Specificity (%)	PPV (%)	NPV (%)
Proposed model	63 (142/227)	72 (46/64)	59(96/163)	41 (46/113)	84 (96/114)

The accuracy, sensitivity, specificity, PPV, NPV, and AUC are all presented as percentages. PPV positive predictive value, NPV negative predictive value, and AUC area under the receiver operating characteristic curve.

Table S4. Summary of model training parameters and performance

Item	YOLOv5 Model Training	ResNet-18Model Training
**Training Data**	4212 images	11452 candidates
**Epochs**	100	10
**Batch Size**	32	8
**Training Devices**	Two Quadro RTX 3090 Ti GPUs	Two Quadro RTX 3090 Ti GPUs
**Actual Training Time**	Approximately 8 hours	Approximately 1 hour 10 minutes

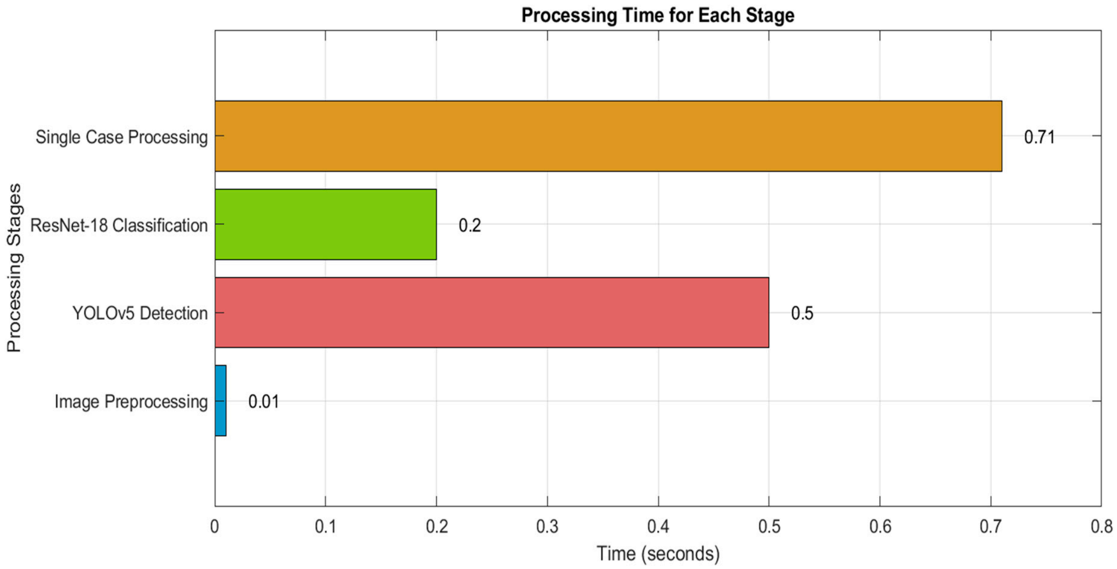

Figure S2. Histogram of processing time for a single data sample.

References

1. Jiang C, Luo Y, Yuan J et al. Ct-based radiomics and machine learning to predict spread through air space in lung adenocarcinoma. Eur Radiol 2020;30:4050-4057.
2. Chen D, She Y, Wang T et al. Radiomics-based prediction for tumour spread through air spaces in stage i lung adenocarcinoma using machine learning. Eur J Cardiothorac Surg 2020;58(1):51-58.
3. Zhuo Y, Feng M, Yang S et al. Radiomics nomograms of tumors and peritumoral regions for the preoperative prediction of spread through air spaces in lung adenocarcinoma. Transl Oncol 2020;13(10):100820.

4. Li C, Jiang C, Gong J, Wu X, Luo Y, Sun G. A ct-based logistic regression model to predict spread through air space in lung adenocarcinoma. Quantitative imaging in medicine and surgery 2020;10(10):1984.
5. Chen L-W, Lin M-W, Hsieh M-S et al. Radiomic values from high-grade subtypes to predict spread through air spaces in lung adenocarcinoma. The Annals of Thoracic Surgery 2022;114(3):999-1006.
6. Onozato Y, Nakajima T, Yokota H et al. Radiomics is feasible for prediction of spread through air spaces in patients with nonsmall cell lung cancer. Sci Rep 2021;11(1):1-10.
7. Liao G, Huang L, Wu S et al. Preoperative ct-based peritumoral and tumoral radiomic features prediction for tumor spread through air spaces in clinical stage i lung adenocarcinoma. Lung Cancer 2022;163:87-95.
8. Bassi M, Russomando A, Vannucci J et al. Role of radiomics in predicting lung cancer spread through air spaces in a heterogeneous dataset. Transl Lung Cancer Res 2022;11(4):560.
9. Takehana K, Sakamoto R, Fujimoto K et al. Peritumoral radiomics features on preoperative thin-slice ct images can predict the spread through air spaces of lung adenocarcinoma. Sci Rep 2022;12(1):1-9.
10. Wang, Yun, et al. "CT-Based Intratumoral and Peritumoral Radiomics Nomograms for the Preoperative Prediction of Spread Through Air Spaces in Clinical Stage IA Non-small Cell Lung Cancer." Journal of Imaging Informatics in Medicine (2024): 1-16.
11. Wang, Shuxing, et al. "CT-Based Super-Resolution Deep Learning Models with Attention Mechanisms for Predicting Spread Through Air Spaces of Solid or Part-Solid Lung Adenocarcinoma." Academic Radiology (2024).
12. Tao J, Liang C, Yin K et al. 3d convolutional neural network model from contrast-enhanced ct to predict spread through air spaces in non-small cell lung cancer. Diagn Interv Imaging 2022;103(11):535-544.
13. Lin, Mong-Wei, et al. "CT-Based Deep-Learning Model for Spread-Through-Air-Spaces Prediction in Ground Glass-Predominant Lung Adenocarcinoma." Annals of Surgical Oncology 31.3 (2024): 1536-1545.
14. Suh, Young Joo, et al. "Computed Tomography Radiomics for Preoperative Prediction of Spread Through Air Spaces in the Early Stage of Surgically Resected Lung Adenocarcinomas." Yonsei Medical Journal 65.3 (2024): 163.
15. Elazab, N., Gab-Allah, W. A., & Elmogy, M. (2024). A multi-class brain tumor grading system based on histopathological images using a hybrid YOLO and RESNET networks. Scientific Reports, 14(1), 4584.
16. Tsuneki, M., & Kanavati, F. (2022). Weakly supervised learning for multi-organ adenocarcinoma classification in whole slide images. Plos one, 17(11), e0275378.
17. Kanavati, F., Ichihara, S., & Tsuneki, M. (2022). A deep learning model for breast ductal carcinoma in situ classification in whole slide images. Virchows Archiv, 480(5), 1009-1022.
18. Lu, W., Toss, M., Dawood, M., Rakha, E., Rajpoot, N., & Minhas, F. (2022). SlideGraph+: Whole slide image level graphs to predict HER2 status in breast cancer. Medical Image Analysis, 80, 102486.

19. Kim, P. J., Hwang, H. S., Choi, G., Sung, H. J., Ahn, B., Uh, J. S., ... & Go, H. (2024). A new model using deep learning to predict recurrence after surgical resection of lung adenocarcinoma. Scientific Reports, 14(1), 6366.
20. Shim, W. S., Yim, K., Kim, T. J., Sung, Y. E., Lee, G., Hong, J. H., ... & Ko, Y. H. (2021). DeepRePath: identifying the prognostic features of early-stage lung adenocarcinoma using multi-scale pathology images and deep convolutional neural networks. Cancers, 13(13), 3308.
21. Wang, C. W., Huang, S. C., Lee, Y. C., Shen, Y. J., Meng, S. I., & Gaol, J. L. (2022). Deep learning for bone marrow cell detection and classification on whole-slide images. Medical image analysis, 75, 102270.
22. Abdeltawab, H. A., Khalifa, F. A., Ghazal, M. A., Cheng, L., El-Baz, A. S., & Gondim, D. D. (2022). A deep learning framework for automated classification of histopathological kidney whole-slide images. Journal of Pathology Informatics, 13, 100093.
23. Ding, K., Zhou, M., Wang, H., Gevaert, O., Metaxas, D., & Zhang, S. (2023). A large-scale synthetic pathological dataset for deep learning-enabled segmentation of breast cancer. Scientific Data, 10(1), 231.
24. Chen, C., Cao, Y., Li, W., Liu, Z., Liu, P., Tian, X., ... & Tian, J. (2023). The pathological risk score: A new deep learning-based signature for predicting survival in cervical cancer. Cancer Medicine, 12(2), 1051-1063.
25. Zhang, S., Zhou, Y., Tang, D., Ni, M., Zheng, J., Xu, G., ... & Zou, X. (2022). A deep learning-based segmentation system for rapid onsite cytologic pathology evaluation of pancreatic masses: A retrospective, multicenter, diagnostic study. EBioMedicine, 80.
26. Zhao, T., Fu, C., Tie, M., Sham, C. W., & Ma, H. (2023). RGSB-UNet: Hybrid Deep Learning Framework for Tumour Segmentation in Digital Pathology Images. Bioengineering, 10(8), 957.
27. Su, F., Zhang, W., Liu, Y., Chen, S., Lin, M., Feng, M., ... & Shen, Y. (2023). The development and validation of pathological sections based U-Net deep learning segmentation model for the detection of esophageal mucosa and squamous cell neoplasm. Journal of Gastrointestinal Oncology, 14(5), 1982.
28. Zhang, S., Yuan, Z., Zhou, X., Wang, H., Chen, B., & Wang, Y. (2024). VENet: Variational energy network for gland segmentation of pathological images and early gastric cancer diagnosis of whole slide images. Computer Methods and Programs in Biomedicine, 250, 108178.
